# Supplementary material for: Association between oral microbiome and seven types of cancers in East Asian population: a two-sample Mendelian randomization analysis
Source: Front Mol Biosci. 2023 Nov 21;10:1327893. doi: 10.3389/fmolb.2023.1327893 (PMC10702768; doi:10.3389/fmolb.2023.1327893)
Supplement: Supplementary file 1 [file DataSheet1.ZIP › Supplementary Materials/MR plots of saliva/saliva═╝/ovarian cancer/pheno.3563_to_ovarian cancer_forest.pdf]

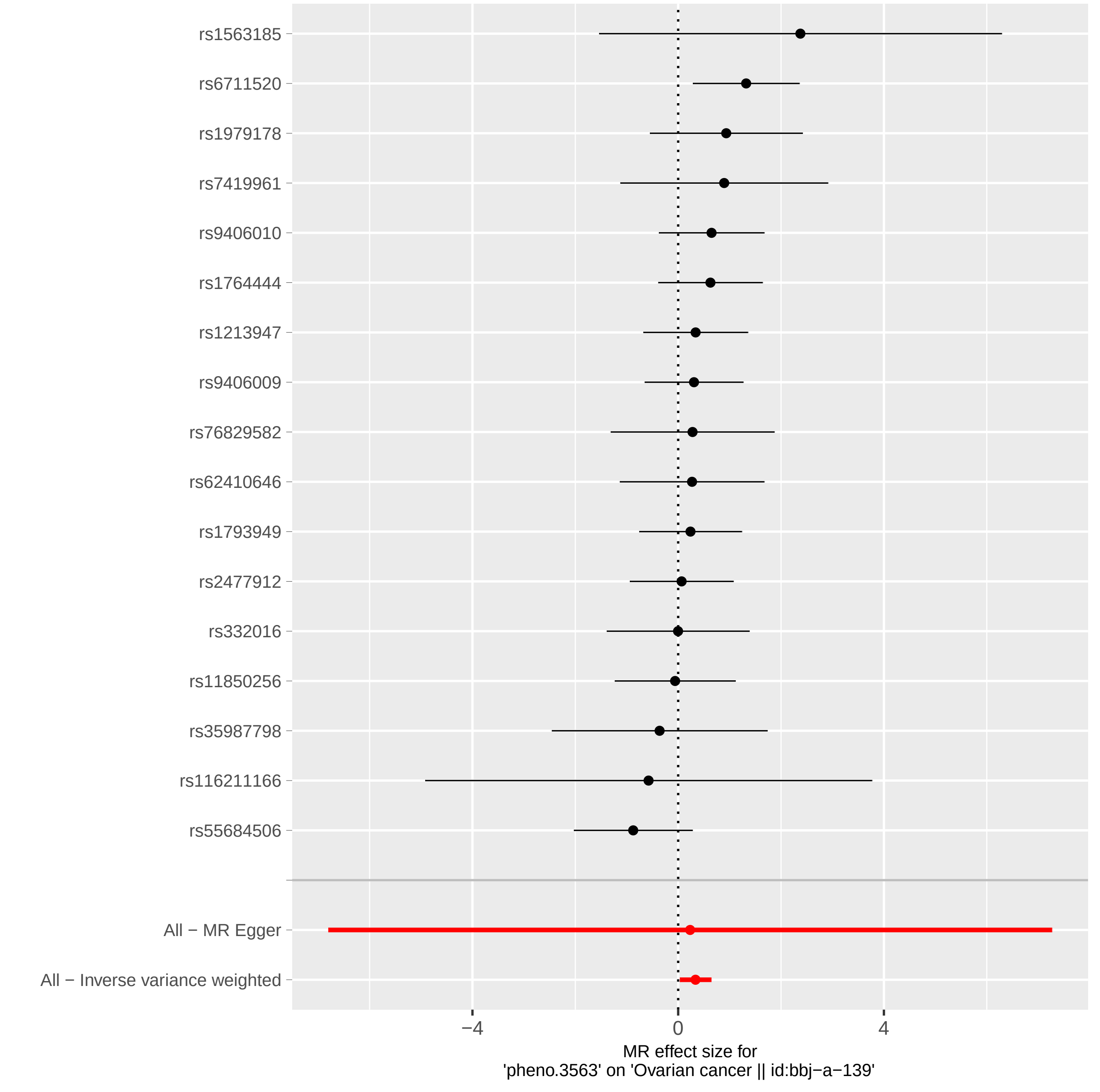

rs1563185
rs6711520
rs1979178
rs7419961
rs9406010
rs1764444
rs1213947
rs9406009
rs76829582
rs62410646
rs1793949
rs2477912
rs332016
rs11850256
rs35987798
rs116211166
rs55684506
All − MR Egger
All − Inverse variance weighted
−4
0
4
MR effect size for
'pheno.3563' on 'Ovarian cancer || id:bbj−a−139'
